# Methodological concerns in the published article in globalization and health: A critical evaluation

**DOI:** 10.1186/s12992-023-01001-z

**Published:** 2023-12-14

**Authors:** Masoud Behzadifar

**Affiliations:** Social Determinants of Health Research Center, Lorestan University of Medical Sciences, Khorramabad, Iran

**Keywords:** Scoping reviews, Database search strategy, Methodological rigor

## Main text

Scoping reviews are vital for research and policy. They systematically map existing literature, highlight gaps, and set research agendas. Methodology is crucial, ensuring transparency and validity by preventing bias, clarifying criteria, and enhancing database searches. Properly conducted, scoping reviews support evidence-based decisions and , guide researchers and policymakers effectively [1]. The article titled “Strategies to Enhance a Climate-Resilient Health System: A Scoping Review,” recently published in the journal Globalization and Health, provides valuable insights for policymakers concerning the adverse impacts of climate change [2]. However, there are significant concerns within the methodology section that need to be addressed.

1. In the legend of Fig. 2, the authors state that it depicts the “PRISMA flow diagram depicting the study selection process.” It is essential to clarify that this flow chart is not a standard PRISMA figure, and it lacks information regarding the reasons for excluding studies and their selection criteria. To align with the PRISMA methodology, all parts of the article should be presented based on this checklist [3].
2. All the authors of this study hold Iranian nationality and they mentioned in the database search section that they searched “The Web of Science, PubMed, Scopus, EMBASE, Scientific Information Database, and Magiran electronic databases and Google Scholar search engine.” Notably, Scientific Information Database [4] and Magiran [5] electronic database are Iranian. The authors also restricted their search to documents and papers in English and Persian languages. This poses a significant challenge as it raises questions about why the authors limited their search to Iranian databases for a global topic. Additionally, it remains unclear why they didn’t include databases in other languages like Spanish, Chinese, Japanese, Arabic, etc. While the study did search four international databases and focused on English language studies, there is no justification for the inclusion of Iranian language databases in the text of the article. Given the global nature of the topic, the concern exists that there is no record of a protocol, and also it is not mentioned in PROSPERO or other review protocol registries. Searching national databases related to the authors’ mother tongue in a scoping review on a global issue can lead to several problems as follows:
  I. Narrowing the global scope: Using national databases may lead to a limited focus on information available in their own country, overlooking important data published in other countries in local languages.
  II. Spatial bias: Relying on national databases may introduce spatial bias, unintentionally emphasizing information from one’s own country and potentially skewing the results.
  III. Incomplete picture: National databases may have limited access to global-level opinions and materials, resulting in an incomplete analysis and presentation of the material.
  IV. Neglecting other languages: Focusing on national databases may cause the authors to overlook valuable research and resources in non-English languages, missing out on significant contributions.
  V. Misrepresentation of diversity: Relying on national databases may misrepresent diversity and homogeneity in the study, providing an inaccurate global perspective.
3. The methodology of the above-mentioned published article does not provide information about eligibility criteria, including publication status, language, years considered, and lacks a rationale for these criteria.
4. Table 1 displays the database search strategy. Notably, the strategy for Magiran database is written in English, while it lacks an English language filter. This introduces bias into a global study.
5. In Table 1, the search strategies for different databases vary. For example, in PubMed, studies are limited to English and Persian languages, while Web of Knowledge limits studies to English only. In Scopus, studies are limited to English and Persian languages, and there are no language restrictions in EMBASE. Scientific Information Database and Magiran seem to focus only on Persian studies. The inconsistency in keyword usage across different databases raises concerns about unintentional bias in the search strategy.
6. The article lacks a section dedicated to data extraction, with the authors mentioning the use of a data extraction form that includes specific data points. However, there is no reference to the specifications of the final studies included in the analysis, which could have been included in an appendix due to the high number of studies analyzed.

My examination of the methodology section underscores the critical need for adhering to validated checklists, such as the PRISMA-ScR (PRISMA Extension for Scoping Reviews) or PRISMA (Preferred Reporting Items for Systematic Reviews and Meta-Analyses), when conducting scoping reviews. By aligning with established frameworks like PRISMA, researchers can ensure transparency, consistency, and rigor in their review processes, ultimately enhancing the credibility of their findings. Registering review protocols in databases like PROSPERO provides a vital roadmap for conducting systematic and scoping reviews, clarifying eligibility criteria, search strategies, and data extraction methods. This registration not only enhances the study’s methodological transparency but also prevents duplication of efforts in the research community. The inclusion of detailed information from selected studies directly within the article’s text is crucial. This approach allows readers to access the specific details of the results, providing a comprehensive understanding of the review’s findings. It also enhances the study’s transparency and supports evidence-based decision-making. In addition, in the Peer-review stage of manuscripts, it is necessary to select and attract reviewers with experience in publishing and conducting review studies. These reviewers can provide valuable insights, ensure methodological rigor, and enhance the overall quality of the review. Given the widespread use of systematic review studies in guiding research and policy development, authors should diligently choose and implement suitable methodologies. This choice impacts the integrity and usefulness of their research, making it imperative to align with validated checklists, register protocols, incorporate detailed study information, and engage experienced referees. These steps collectively contribute to the credibility and reliability of scoping reviews and, in turn, empower researchers and policymakers in addressing critical global questions and advancing our understanding of complex issues.

## Data Availability

Not applicable.

## Abbreviations

PRISMA-ScR: PRISMA Extension for Scoping Reviews
PRISMA: Preferred Reporting Items for Systematic Reviews and Meta-Analyses
MOOSE: Meta-analysis Of Observational Studies in Epidemiology
STROBE: Strengthening the Reporting of Observational Studies in Epidemiology
CONSORT: Consolidated Standards of Reporting Trials
